# A combined DHA-rich fish oil and cocoa flavanols intervention does not improve cognition or brain structure in older adults with memory complaints: results from the CANN randomized, controlled parallel-design study

**DOI:** 10.1016/j.ajcnut.2023.06.008

**Published:** 2023-06-12

**Authors:** David Vauzour, Andrew Scholey, David J White, Neal J Cohen, Aedín Cassidy, Rachel Gillings, Michael A Irvine, Colin D Kay, Min Kim, Rebecca King, Cristina Legido-Quigley, John F Potter, Hilary Schwarb, Anne-Marie Minihane

**Affiliations:** 1 Norwich Medical School, University of East Anglia (UEA), Norwich, United Kingdom; 2 Centre for Human Psychopharmacology, Swinburne University, Australia; 3 Beckman Institute for Advanced Science and Technology, University of Illinois at Urbana-Champaign, Urbana, IL, United States; 4 Institute for Global Food Security, Queen’s University Belfast, Belfast, Northern Ireland; 5 Plants for Human Health Institute, Food Bioprocessing and Nutrition Sciences Department, North Carolina State University, North Carolina Research Campus, Kannapolis, NC, United States; 6 Translational and Clinical Chemistry, Kings College London, London, Norwich, United Kingdom; 7 Norwich Institute of Healthy Ageing (NIHA), UEA, Norwich, Norwich, United Kingdom

**Keywords:** omega-3, polyphenols, flavonoids, mild cognitive impairment, subjective cognitive impairment, aging, cognition

## Abstract

**Background:**

There is evidence that both omega-3 long-chain polyunsaturated fatty acids (PUFAs) (eicosapentaenoic acid [EPA] and docosahexaenoic acid [DHA]) and cocoa flavanols can improve cognitive performance in both healthy individuals and in those with memory complaints. However, their combined effect is unknown.

**Objectives:**

To investigate the combined effect of EPA/DHA and cocoa flavanols (OM3FLAV) on cognitive performance and brain structures in older adults with memory complaints.

**Methods:**

A randomized placebo-controlled trial of DHA-rich fish oil (providing 1.1 g/d DHA and 0.4 g/d EPA) and a flavanol-rich dark chocolate (providing 500 mg/d flavan-3-ols) was conducted in 259 older adults with either subjective cognitive impairment or mild cognitive impairment. Participants underwent assessment at baseline, 3 mo, and 12 mo. The primary outcome was the number of false-positives on a picture recognition task from the Cognitive Drug Research computerized assessment battery. Secondary outcomes included other cognition and mood outcomes, plasma lipids, brain-derived neurotrophic factor (BDNF), and glucose levels. A subset of 110 participants underwent structural neuroimaging at baseline and at 12 mo.

**Results:**

197 participants completed the study. The combined intervention had no significant effect on any cognitive outcomes, with the exception of reaction time variability (P = 0.007), alertness (P < 0.001), and executive function (P < 0.001), with a decline in function observed in the OM3FLAV group (118.6 [SD 25.3] at baseline versus 113.3 [SD 25.4] at 12 mo for executive function) relative to the control, and an associated decrease in cortical volume (P = 0.039). Compared with the control group, OM3FLAV increased plasma HDL, total cholesterol ratio (P < 0.001), and glucose (P = 0.008) and reduced TG concentrations (P < 0.001) by 3 mo, which were sustained to 12 mo, with no effect on BDNF. Changes in plasma EPA and DHA and urinary flavonoid metabolite concentrations confirmed compliance to the intervention.

**Conclusions:**

These results suggest that cosupplementation with ω-3 PUFAs and cocoa flavanols for 12 mo does not improve cognitive outcomes in those with cognitive impairment.

This trial was registered at clinicaltrials.gov as NCT02525198.

## Introduction

At a population level, interventions that delay the onset of dementia by 2 y are predicted to reduce the number of dementia patients by 20% [1,2]. Prospective cohort studies have consistently reported cognitive and neurophysiological benefits of the fish-derived omega-3 long-chain PUFAs, EPA, and DHA [[3], [4], [5]] and plant-derived flavanols (FLAVs) found in many fruits such as berries and apples but also in cocoa and teas [[6], [7], [8], [9], [10], [11]], with effect sizes equivalent to approximately 4 y of aging in the highest consumers of fish [12] or flavonoids [13], respectively.

Investigations in model systems add validity to the prospective cohort evidence. Furthermore, they provide insights into the molecular and physiological mechanisms underlying long-chain ω-3 PUFAs and FLAVs which suggest that they could confer additive and potentially synergistic effects. Indeed, DHA supplementation increases its availability for neuronal membrane synthesis, neurite outgrowth, and neurogenesis and promotes synaptic plasticity [14,15]. FLAV exposure enhances neuronal survival, spine density formation, the production of brain-derived neurotrophic factor (BDNF), is insulin sensitizing, and positively modulates brain function [[16], [17], [18], [19], [20]]. Both compounds are anti-inflammatory via complementary pathways [21,22]. Finally, ω-3 PUFAs and FLAVs may improve cognition through improved vascular function and cerebral blood flow [[18], [19], [20],^23^].

However, randomized controlled trials that have intervened with either EPA/DHA or flavonoids are conflicting, with many showing marginal or no meaningful cognitive benefits [[24], [25], [26]]. We designed the Cognitive Ageing, Nutrition and Neurogenesis (CANN) clinical trial to test the effect of combined supplementation with ω-3 long-chain PUFAs and FLAVs on cognitive function in adults with some evidence of memory decline. This study hypothesized that 12-mo administration of a combination of 500 mg cocoa FLAVs with 1.5 g ω-3 long-chain PUFAs would improve cognitive function in a mixed subjective cognitive impairment (SCI) and mild cognitive impairment (MCI) cohort. Such individuals are at increased risk of dementia, and there is a growing consensus that neurological changes underlying Alzheimer’s disease (AD) may typically begin several decades before their overt behavioral manifestation in AD [27]. Commencing interventions in at-risk cohorts may provide the best chance of mitigating against cognitive decline and dementia. The inclusion of both SCI and MCI (ie, encompassing those without and with objective cognitive impairment, respectively) increased the chances that any positive effects could be translated.

The primary efficacy endpoint was hippocampal dentate gyrus-sensitive cognitive function, assessed by the number of false-positives on the Picture Recognition Task of the Cognitive Drug Research (CDR) computerized assessment system. The latter is analogous to the classic pattern separation paradigm, which is highly dependent on hippocampal function [28]. In addition, the iPosition paradigm was included as a measure of relational memory, which has also demonstrated high sensitivity to hippocampal integrity as reported previously [29,30]. This study focused on hippocampal function for several reasons. First, the hippocampus is critically involved in cognitive processes, which decline with aging [31]. Second, there are different levels of hippocampal atrophy that, from most to least pronounced, differentiate AD, MCI, SCI, and normal aging cognitive phenotypes [32]. Third, unlike other brain regions, the human hippocampus shows nonlinear atrophy from the sixth decade of life [33]. Fourth, the hippocampus is 1 of only 2 brain loci that may display adult neurogenesis [30]. Finally, the rate of hippocampal atrophy is sensitive to lifestyle factors including dietary factors [3,34]. Indeed, previous research has reported that cocoa FLAV administration increases dentate gyrus perfusion [18], which may be a proxy for hippocampal neurogenesis.

### Subjects and Methods

The study protocol and methods have been described in detail elsewhere [35].

### Ethics

The conduct, evaluation, and documentation of this study abide by Good Clinical Practice guidelines and the guiding principles of the Declaration of Helsinki. The study was approved by the Bellberry Human Research Ethics Committee (Study ID: 2015-03-227) and Swinburne University Human Research Ethics Committee (SHR Project 2015-208) for the Swinburne University of Technology (SUT) site and the National Research Ethics Service Committee (Study ID: 14/EE/0189) for the University of East Anglia (UEA) site. All participants provided informed signed consent prior to participating. The trial was registered at clinicaltrials.gov as NCT02525198.

### Study design and participants

The CANN trial was a 12-mo randomized placebo-controlled parallel study with 2 intervention arms. There were 2 recruitment sites, the UEA (Norwich, UK) and SUT (Melbourne, Australia), with MRI analyzed centrally at the University of Illinois (Urbana Champaign, Urbana, IL, United States). Participants were asked to attend 3 clinical visits, at 0 mo (baseline), 3 mo, and 12 mo, to undergo cognitive assessment and provide clinical measures and biological samples. MRI assessment was conducted at baseline and 12 mo in a subset of *n =* 110 participants.

Participants recruited were aged ≥55 y, with either SCI or MCI [36] but without a diagnosis or evidence of dementia (Montreal Cognitive Assessment [MoCA] score ≥18), or significant depression (Geriatric Depression Scale-15 score <10) or other neurological disorder as judged by the trial clinician [35]. Those with a BMI ≥40 kg/m^2^, uncontrolled hypertension, a history of MRI evidence of brain trauma, a high flavonoid intake (flavonoid supplement use or >15 portions of flavonoid-rich foods per day defined as any fruits, vegetables, tea, coffee, fruit juice, red wine, cocoa/dark chocolate), EPA/DHA supplement use, high ω-3 fatty acid status (red blood cells EPA+DHA >6% of total fatty acids), or who had a major cardiovascular event in the previous 12 mo were precluded from participating. A full list of inclusion and exclusion criteria has been previously reported [35].

### Intervention, assignment to group, and blinding

Participants were asked to consume 3 × 1 g oil capsules and 33 g of chocolate drops of either test or control products daily with the main meal of the day. The test capsules provided 1.1 g DHA and 0.4 g EPA per day in triglyceride form. The control capsules contained a blend of 80% palm oil and 20% corn oil to deliver a fatty acid composition typical of a UK or Australian diet. Both the test and control capsules contained 1% lemon oil, to maintain study blinding, and 1% mixed tocopherols for stability of the study oils. The test chocolate provided 508 mg of total flavan-3-ols ranging from monomers to decamers (ie, degrees of polymerization from 1 to 10) (Supplementary Table 1). The control chocolate drops were isocaloric, generally matched for macronutrient composition but not for methylxanthines, and delivered 38 mg flavan-3-ols. Both sets of capsules and chocolate products presented with similar sensorial characteristics and were in identical packaging.

Assignment to the test or control groups was conducted using a randomization algorithm (Covariate Adaptive Randomization software), with groups stratified for *APOE* genotype (E4 carriers [E3/E4 and E4/E4] compared with non-E4 carriers [E2/E2, E2/E3, E3/E3]; Supplementary Table 2), sex, and cognitive status (SCI compared with MCI). The trial researchers, clinical staff, statistician (KW) and participants were all blinded to group (test or control assignment). Adherence to intervention was assessed by analysis of EPA and DHA in the plasma lysophosphatidylcholine (LPC) and select PC fraction, along with 3 flavan-3-ol metabolites in urine, and by return of empty chocolate sachets and oil capsule bottles. The threshold for categorization as adherent to the intervention based on the latter was set at 80%.

### Cognitive outcomes

As described previously, cognition was assessed at each clinical visit using the CDR computerized assessment system (Bracket Cognition), the MoCA, the Delis–Kaplan Executive Function System, the Verbal Fluency Test (0 and 12 mo only), and the iPosition task. Paper-and pencil Bond-Lader Visual Analogue Scale of Mood and Alertness along with the Leeds Sleep Evaluation Questionnaire were also employed.

The CDR test battery [37] has shown sensitivity to cognitive changes, including in trials of MCI, dementia, and SCI. It has been used in over 1200 clinical trials across 3000 sites in more than 60 countries. Tasks are presented via color monitors on laptops, with the exception of the written Word Recall tasks, which are performed with a pen and paper. All responses were recorded via a 2-button (YES/NO) response box. The entire battery took approximately 25 to 30 min to complete. The following CDR tasks were completed (in order): Word Presentation, Immediate Word Recall, Picture Presentation, Simple Reaction Time, Digit Vigilance, Choice Reaction Time, Spatial Working Memory, Numeric Working Memory, Delayed Word Recall, Word Recognition, and Picture Recognition Task. Five composite measures of cognitive ability can then be derived from these tasks, as follows: Power (speed) of Attention, Continuity (accuracy) of Attention, Quality of Working Memory, Quality of Episodic Secondary Memory, and Speed of Memory. The primary efficacy endpoint was hippocampus dentate gyrus-sensitive cognitive function, assessed by the number of false-positives on the Picture Recognition Task of the CDR [38]. Details on how the cognitive composite scores were calculated can be found here [39]. The iPosition paradigm, as previously described [33], is a measure of relational memory which has also demonstrated high sensitivity to hippocampal integrity [29]. It can be used to measure deficits in relational memory in patients with mild hippocampal damage, even when standardized neuropsychological measures of hippocampal-based memory fail to do so [34].

### MRI

A subset of participants, representative of the full group, at both trial sites completed a neuroimaging session at baseline and 12 mo. Here, we report volumetric measures of brain structure in this neuroimaging subset derived from T1-weighted anatomical scans acquired at both sites. Further imaging modalities acquired in this neuroimaging subset are to be reported elsewhere and are detailed in Appendix F of the trial protocol paper [35]. At the UEA site, T1-weighted 3D gradient-echo sequences were acquired with a Discovery MR750w 3T instrument (General Electric) using a 3D fast spoiled gradient-echo brain volume imaging sequence in the sagittal orientation (repetition time/echo time/inversion time = 7,040/2.612/900 ms, 0.9 mm isotropic resolution, field of view = 230 × 230 mm, number of excitations = 0.5). At the SUT site, the T1-weighted structural images were acquired using a Siemens TIM Trio 3T system via a 3D magnetization-prepared rapid gradient-echo sequence (repetition time/echo time/inversion time = 1900/2.32/900 ms, 0.9 mm isotropic resolution, field of view = 230 × 230 mm, generalized autocalibrating partial parallel acquisition acceleration factor of 2). At both sites, data were collected using 32-channel head coils.

T1-weighted images were processed following the longitudinal processing pipeline [40] of FreeSurfer (Version 5.3 [41]). The longitudinal pipeline increases reliability of volumetric estimates by using an unbiased template [42,43] to initialize standard FreeSurfer processing steps, including skull stripping, Talairach transformation, atlas registration, spherical surface maps, and parcellations. Results of the recon-all pipeline were inspected and manual editing conducted where necessary at each stage of the processing, first on cross-sectional results of each timepoint, then the template, and finally on the longitudinal output (https://surfer.nmr.mgh.harvard.edu/fswiki/LongitudinalEdits). Total hippocampal (left + right hippocampus) was the preregistered secondary outcome for structural MRI measures, and ventricular (Left Lateral Ventricle + Left Inferior Lateral Ventricle + Right Lateral Ventricle + Right Inferior Lateral Ventricle), cortical, and white matter volumes (mm^3^), as exploratory measures, were then extracted for analysis, with the estimated total intracranial volume also used in the statistical models of volumetric analyses as a covariate.

### Biological sample collection and analysis

Participants provided an overnight fasted blood sample and a spot urine sample that was their first void of the day.

For FLAV and methylxanthine analyses, the metabolites were purified from 50 μL human urine by 96-well plate solid phase extraction (Strata™-X Polymeric Reversed Phase, microelution 2 mg/well). The solid phase extraction treated samples were chromatographically separated and quantified using Exion high-performance ultrahigh performance LC-tandem MS SCIEX QTRAP 6500+ enhanced ultrahigh performance hybrid triple quadrupole-linear ion trap MS with electrospray IonDrive Turbo-V Source. The samples were injected into a Kinetex PFP UPLC column (1.7 μm particle size, 100 Å pore size, 100 mm length, 2.1 mm internal diameter; Phenomenex) with oven temperature maintained at 37°C. Mobile phase A and mobile phase B consisted of 0.1% v/v formic acid in water (Optima grade, Fisher Scientific) and 0.1% v/v formic acid in LC-MS grade acetonitrile (Honeywell Burdick and Jackson) respectively, with a binary gradient ranging from 2% B to 90% B over 30 min and flow rate gradient from 0.55 mL/min to 0.75 mL/min. MS/MS scanning was accomplished by advanced scheduled multiple reaction monitoring using polarity switching between positive and negative ionization mode in Analyst (v.1.6.3, SCIEX) and with quantitation conducted using MultiQuant (v.3.0.2, SCIEX) software platforms. Internal standards included L-tyrosine-^13^C9,^15^N, resveratrol-(4-hydroxyphenyl-^13^C6, and phlorizin dehydrate (Sigma-Aldrich), and 11-point calibration curves (1 nM to 10 μM) were established for 10 compounds whereas 12-point calibration curves (1 nM to 25 μM) were required for 2 compounds. Calibration curves were made by spiking reference standards in matrix matched solid phase extracted Surine which is alternative of human urine (synthetic urine negative control; S-020, Sigma-Aldrich Corporation). Source parameters included: curtain gas 35, ion-spray voltage 4000, temperature 550, nebulizer gas 70, and heater gas 70. The optimized analyte specific quadrupole voltages (mean ± SD) for negative mode were 50 ± 19 for declustering potential, 9 ± 2 for entrance potential, 26 ± 05 for collision energy, and 11 ± 6 for collision cell exit potential. Similarly, for positive mode, the optimized analyte specific quadrupole voltages (mean ± SD) were 29 ± 0 for declustering potential, 6 ± 0 for entrance potential, 29 ± 12 for collision energy, and 12 ± 4 for collision cell exit potential.

The targeted metabolite analysis protocol was optimized and validated to detect 43 analytes, which were quantified relative to 12 authentic commercial and synthetic standards. Where reference standards for metabolites (including FLAV Phase II conjugates) were not available (31 analytes), identification was based on fragmentation profiling involving the precursor structure and 3 to 5 product transitions and confirmed in pooled samples. These metabolites were then quantified relative to their closest structural reference standard with similar ionization intensities (Supplementary Table 3). Finally, all metabolites were confirmed on the basis of established retention times (using authentic and synthesized standards where possible) and 3 or more precursor-to-product ion transitions.

For the fatty acid analysis, plasma samples were analyzed for AA, EPA, and DHA using free/NEFA and lysophosphatidylcholine (LPC) fractions. NEFA and LPC were chosen because they are quantitatively the most important and efficient respectively at delivering DHA to the brain, across the blood brain barrier [44]. Phosphatidylcholine (PC), PC 38:5, PC 38:6, and PC 40:6, were also measured as EPA- and DHA-containing PC molecules. Ten microliters of high purity water and 40 μL of MS-grade methanol containing internal standards (pentadecanoic acid in NEFA and LPC forms) were added to 20 μL of plasma followed by a 2-min vortex mix to precipitate proteins. Two hundred microliters of methyl t-butyl ether was added, and the samples were mixed via vortex at room temperature for 1 h. After the addition of 50 μL of high purity water, a final sample mixing was performed before centrifugation at 10,000 g for 10 min. The upper, lipid-containing, methyl t-butyl ether phase was then extracted and analyzed by LC-MS (Q-Exactive Plus, Thermo, MA, USA) in single ion monitoring mode to increase sensitivity. The analytical method is detailed elsewhere [45]. The single molecule integrated peak areas under the exact mass chromatograms (representing the levels of the lipid) were obtained by Skyline [46].

BDNF was quantified using a commercially available ELISA assay in serum samples, according to the manufacturer’s instructions (ab212166 – Human BDNF SimpleStep ELISA Kit, Abcam).

### Dietary assessment

Assessment of diet at baseline and 12 mo was conducted using a 130-item The European Prospective Investigation into Cancer and Nutrition FFQ [35]. Nutrient intake was established from the FFQ data using assigned nutrient values from the seventh edition of McCance and Widdowson food composition tables [47], with flavonoid intakes established using the USDA flavonoid database [48].

### Statistical analyses and sample size estimation

The sample size estimate has been detailed previously [35]. It is based on the predicted impact of a combination of 1.5 g long-chain ω-3 PUFA (1.1 g DHA, 0.4 g EPA) and 500 mg cocoa flavan-3-ols (OM3FLAV) administered daily for 12 mo, which has not been used previously in an intervention study. Based on the range of the individual effect sizes, we conservatively based our power calculation on a medium effect size, ie, Cohen’s d of approximately 0.5, which generates a sample size of 108/group for a 2-arm trial, with 90% power to detect a significant change at the 5% probability level. A recruitment target of 120 randomized per intervention assumed a 10% attrition rate. The sample size calculation is fully articulated in our protocol paper [35].

All non-MRI analyses were conducted by ANCOVA using mixed models for repeated measures with SAS PROC MIXED. INTERVENTION was fitted as a fixed between-group factor (2 levels: intervention or control group). For non-MRI data, change from baseline was calculated for all outcome variables (ie, 3 mo – 0 mo and 12 mo – 0 mo), and VISIT was fitted as a fixed repeated factor (2 levels: 3 and 12 mo). For MRI data, the 0-mo and 12-mo absolute data was fitted as a 2-level repeated factor. INTERVENTION×VISIT interaction was fitted to the model. The Kenward-Roger approximation was used to estimate denominator degrees of freedom. The LSMEANS statement was used to perform pairwise comparisons between the 2 intervention groups, both overall and at each individual time point. The LSMeans estimates were calculated together with the *t* and *P* values from each comparison.

BASELINE (predose scores) and study site were fitted as covariates. Additional potential covariates included, MCI/SCI status, *APOE4* carrier status, sex, years of education, intelligence quotient, age, and BMI, with only years of education and BMI differing between the intervention groups at baseline and included as covariates in the final model. Further predefined exploratory analysis was conducted to determine the impact of sex, *APOE4* carrier status, and cognitive status (SCI compared with MCI) on the response to intervention of the primary endpoint.

**FIGURE 1:**
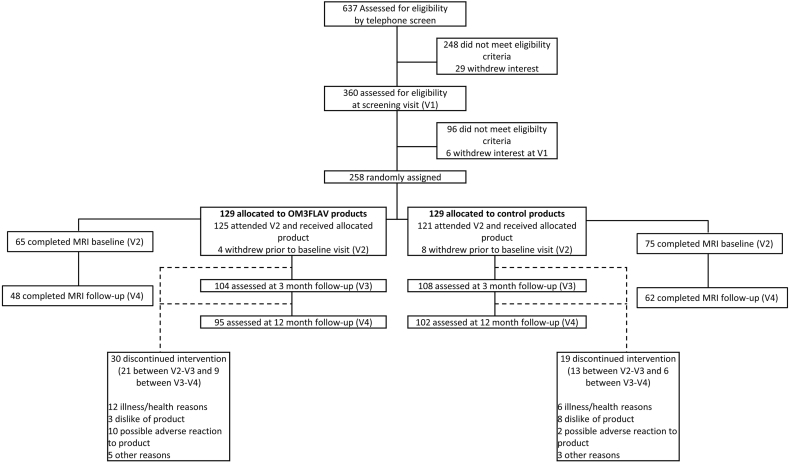
CONSORT flowchart diagram. A total of *N =* 258 participants were randomly assigned to either a DHA-rich fish oil and cocoa flavanol mixture (OM3FLAV; *n =* 129) or a control product (*n =* 129).

For the MRI analysis, estimated total intracranial volume, education attainment, and center were added as covariates to the model. The MRI data was evaluated according to the change from an initial baseline scan, thereby overcoming any heterogeneity introduced into the data due to differences in the instruments used at the study sites. Postprocessing of the MRI measures obtained at both sites was conducted at the Biomedical Imaging Center (University of Illinois) to ensure consistency across sites. The effect of group was determined by a univariate ANOVA on the absolute change from 0 to 12 mo (SPSS Version 28.0.1.0). All participants selected for the main per protocol (PP) analyses had completed the entire study and were free of major protocol violations (including medication violations and an overall intervention product compliance of <80%). As a supportive analysis, an intention-to-treat analysis was conducted including all subjects who received at least 1 dose of the study intervention and had postintervention data available. This analysis included *N =* 212 participants who attended the 3 mo clinical visit (Figure 1).

SAS software (STAT 15.1) was used for all statistical analyses

## Results

### Participant characteristics

A total of 637 individuals were assessed for eligibility, with 258 participants recruited and randomized to control or test intervention; 246 completed the baseline measurements, and 125 participants were randomized into the active OM3FLAV intervention group and 121 into the control group. There was no difference in age, years of education, blood pressure, or global cognitive performance as assessed by the MoCA at baseline. Overall, 57% were females and 29% were *APOE4* carriers, with a SCI/MCI split of 142/104 (Table 1). One hundred and ninety-seven participants completed the trial. This presented a drop-out rate of 19.9%, with 24% in the test and 16% in the control group. The main reasons for discontinuing were health reasons (*n =* 18), dislike of the intervention products (*n =* 11), possible minor adverse reaction to the products (*n =* 12), and other reasons (*n =* 8) (see Figure 1 for detail). The means for the drop-out group were comparable to the completers for age (mean 65.4 y compared with 65.4 y), BMI (27.5 kg/m^2^ compared with 26.7 kg/m^2^), MoCA score (26.5 compared with 27.1), and years of education (14.7 y compared with 14.5 y). Of the dropouts, 51% and 49% were SCI and MCI respectively. The *APOE4* carrier status was comparable between the group as a whole at baseline (Table 1, *n =* 246) and participants who dropped out (*n =* 49), at 29% and 31% respectively. Interestingly, 37/49 (76%) of the dropouts were female compared with 57% female at baseline. The trial CONSORT Flow Diagram is provided in Figure 1.

**TABLE 1:** Baseline characteristics of the intention-to-treat population

	Overall (*N =* 246)	Control (*N =* 121)x	OM3FLAV (*N =* 125)
**Age (y)**	65.5 (6.5)	65.0 (6.7)	66.0 (6.3)
**Sex, M/F (%F)**	107/139 (57)	51/70 (59)	56/69 (55)
**Education (y)**	14.5 (3.7)	14.1 (3.2)	14.9 (4.2)
**SCI/MCI (%MCI)**	142/104 (42)	71/50 (41)	71/54 (43)
**MoCA score**^1^	27.0 (2.3)	27.1 (2.3)	27.0 (2.3)
**% *APOE4* (*n =* 241) *n*, (%)**^2^	69 (29)	34 (29)	35 (28)
**BMI (kg/m**^**2**^**)**	26.9 (4.3)	26.6 (4.5)	27.2 (4.2)
**Blood pressure (mmHg)**			
Systolic	139 (15)	139 (15)	138 (14)
Diastolic	81 (9)	80 (9)	81 (9)
Omega-3 index (%)	4.7 (0.9)	4.7 (0.8)	4.7 (1.0)
**Medication use**			
Antihypertensives (*n =* 231)	54 (23)	24 (21)	30 (25)
Statins (*n =* 231)	37 (16)	18 (16)	19 (16)
Antidepressants (*n =* 231)	15 (6)	8 (7)	7 (6)
HRT (*n =* 139 females)	14 (10)	6 (9)	8 (12)

Data are mean (SD) or as stated *n* (%).

BDNF, brain-derived neurotrophic factor; MCI, mild cognitive impairment; MoCA, Montreal Cognitive Assessment; Omega-3 index, eicosapentaenoic acid plus docosahexaenoic acid as a % of total fatty acids, in red blood cells; OM3FLAV, omega-3 polyunsaturated fatty acids (EPA+DHA; OM3) + cocoa flavan-3-ols (FLAV); SCI, subjective cognitive impairment.

1 *n =* 2 participants had a MoCA score <20 at baseline

2 *APOE4* carriers (E3/E4, E4/E4) versus non-*APOE4* carriers (E2/E2, E2/E3, E3/E3)

Based on packaging return, for those who attended the 12 mo clinical visit, compliance to intervention was 95.5% and 95.1% in the control and OM3FLAV groups, respectively. Two individuals violated protocol due to medication use and therefore were excluded from the PP analysis. The final PP analysis included *n =* 122 from the Norwich and *n =* 73 from the Melbourne sites.

### n-3 PUFA and FLAV response to intervention

**FIGURE 2:**
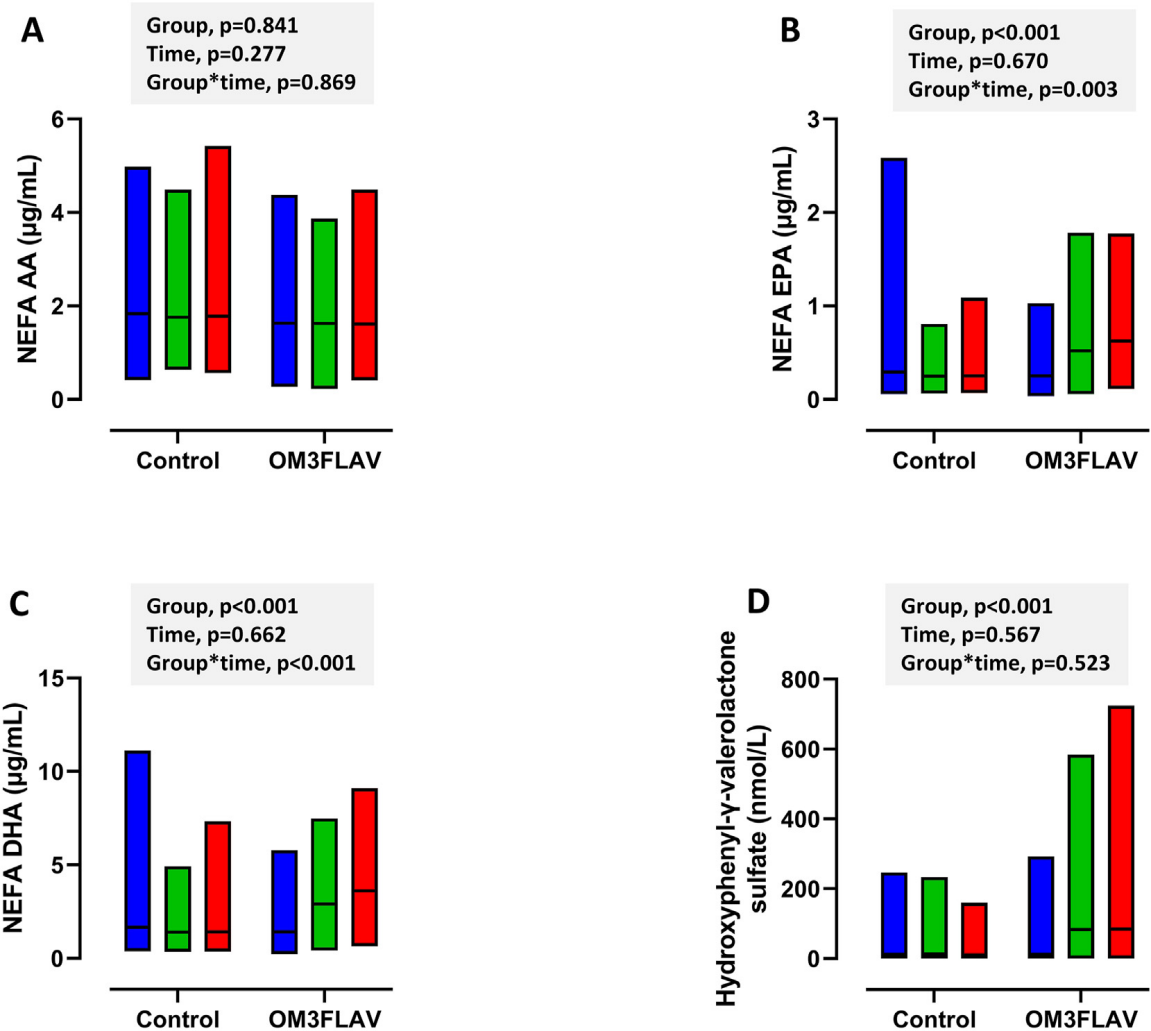
Impact of 3-mo and 12-mo consumption of a DHA-rich fish oil and cocoa flavanol mixture (OM3FLAV; *n =* 104 at 3 mo and *n =* 95 at 12 mo) or a control product (*n =* 108 at 3 mo and *n =* 102 at 12 mo) on plasma fatty acid profiles and urinary excretion of a flavanol metabolite. (A) Non-esterified AA (NEFA AA); (B) Non-esterified eicosapentaenoic acid (NEFA EPA); (C) Non-esterified docosahexaenoic acid (NEFA DHA); (D) Hydroxyphenyl-γ-valearolactone sulfate. Data are presented as floating bars (min-max) with the horizontal line indicating the mean. Bar colors are as follows: blue (baseline), green (3 mo), and red (12 mo).

As anticipated, OM3FLAV intervention resulted in a significant increase in NEFA EPA and DHA and LPC EPA and DHA, along with PC 38:5, PC 38:6, and PC 40:6 fractions (*P <* 0.001), with 2 to 3 times higher concentrations at 3 mo and 12 mo relative to baseline (Table 2). For the NEFA only fraction, a time×group interaction was only evident for EPA and DHA, with further increases between 3 mo and 12 mo (Figure 2B, C). A total of 43 flavanol metabolites were quantified in the urine samples with 3, namely 5-(phenyl)-γ-valerolactone-3'-sulfate, hydroxyphenyl-γ-valerolactone-sulfate and 5-(3'-hydroxyphenyl)-γ-valerolactone-4'-*O*-glucuronide (Table 2 and Figure 2D) reported here for compliance. OM3FLAV increased the concentration of all 3 metabolites, 7 to 10 times, with no change following the control intervention. For the FLAV metabolites, peak excretion rates were reached by 3 mo with no time×group interactions evident (*P =* 0.073-0.523).

**TABLE 2:** Plasma n-3 PUFA, and urinary flavonoids at baseline and in response to intervention

	0 mo	3 mo	12 mo	*P*_group	*P*_time	*P*_group×time
**LPC AA (μg/mL)**						
Control (*n =* 62)	7.84 (7.88)	7.85 (7.67)	7.82 (7.23)	<0.001	0.857	0.348
OM3FLAV (*n =* 59)	7.66 (6.62)	6.73 (6.19)	6.27 (5.01)			
**LPC EPA (μg/mL)**						
Control (*n =* 62)	2.63 (2.803)	2.71 (2.75)	2.72 (2.82)	<0.001	0.320	0.502
OM3FLAV (*n =* 59)	2.62 (2.80)	3.56 (3.35)	3.43 (2.81)			
**LPC DHA (μg/mL)**						
Control (*n =* 62)	1.72 (1.70)	1.78 (1.91)	1.78 (1.78)	<0.001	0.632	0.757
OM3FLAV (*n =* 59)	1.75 (1.52)	3.48 (3.24)	3.37 (2.75)			
**PC 36:5 (μg/mL)**						
Control (*n =* 98)	50.9 (19.4)	51.4 (16.7)	51.6 (15.9)	<0.001	0.157	0.789
OM3FLAV (*n =* 89)	53.2 (20.1)	102.1 (16.7)	102.8 (29.7)			
**PC 38:6 (μg/mL)**						
Control (*n =* 98)	69.6 (15.5)	68.1 (15.0)	67.5 (13.5)	<0.001	0.810	0.658
OM3FLAV (*n =* 89)	69.7 (14.8)	124.3 (21.7)	124.7 (24.2)			
**PC 40:6 (μg/mL)**						
Control (*n =* 98)	18.2 (4.9)	17.9 (5.0)	17.7 (4.4)	<0.001	0.677	0.198
OM3FLAV (*n =* 89)	17.9 (4.4)	37.7 (7.6)	38.5 (8.3)			
**5-(Phenyl)-γ-valerolactone-3'-sulfate (nmol)**						
Control (*n =* 100)	4.1 (32.7)	5.5 (31.7)	4.9 (19.2)	<0.001	0.689	0.073
OM3FLAV (*n =* 92)	3.91 (6.1)	39.9 (56.3)	29.9 (48.3)			
**5-(3'-Hydroxyphenyl)-γ-valerolactone-4'-*O*-glucuronide (nmol)**						
Control (*n =* 99)	12.2 (33.6)	14.0 (32.5)	10.6 (20.3)	<0.001	0.568	0.523
OM3FLAV (*n =* 89)	10.8 (33.3)	74.7 (111.4)	78.8 (140.3)			

Data are mean (SD); ANCOVA using mixed models for repeated measures, with change from baseline (i.e. 3 mo-0 mo and 12 mo-0 mo) fitted as a fixed repeated factor (2 levels: 3 and 12 mo), and center, age, sex and BMI added as covariates.

LPC, lysophosphatidylcholine; PC, phosphatidylcholine, PC.36:5 (16:0/20:5, 16:1/20:4, 18:2/18:3), PC.38:6 (16:0/22:6, 18:1/20:5, 18:2/20:4), PC.40:6 (18:0/22:6, 18:1/22:5, 18:2/22:4, 20:2/20:4); OM3FLAV, omega-3 polyunsaturated fatty acids (EPA+DHA; OM3) + cocoa flavan-3-ols (FLAV).

### Primary outcome measure

**FIGURE 3:**
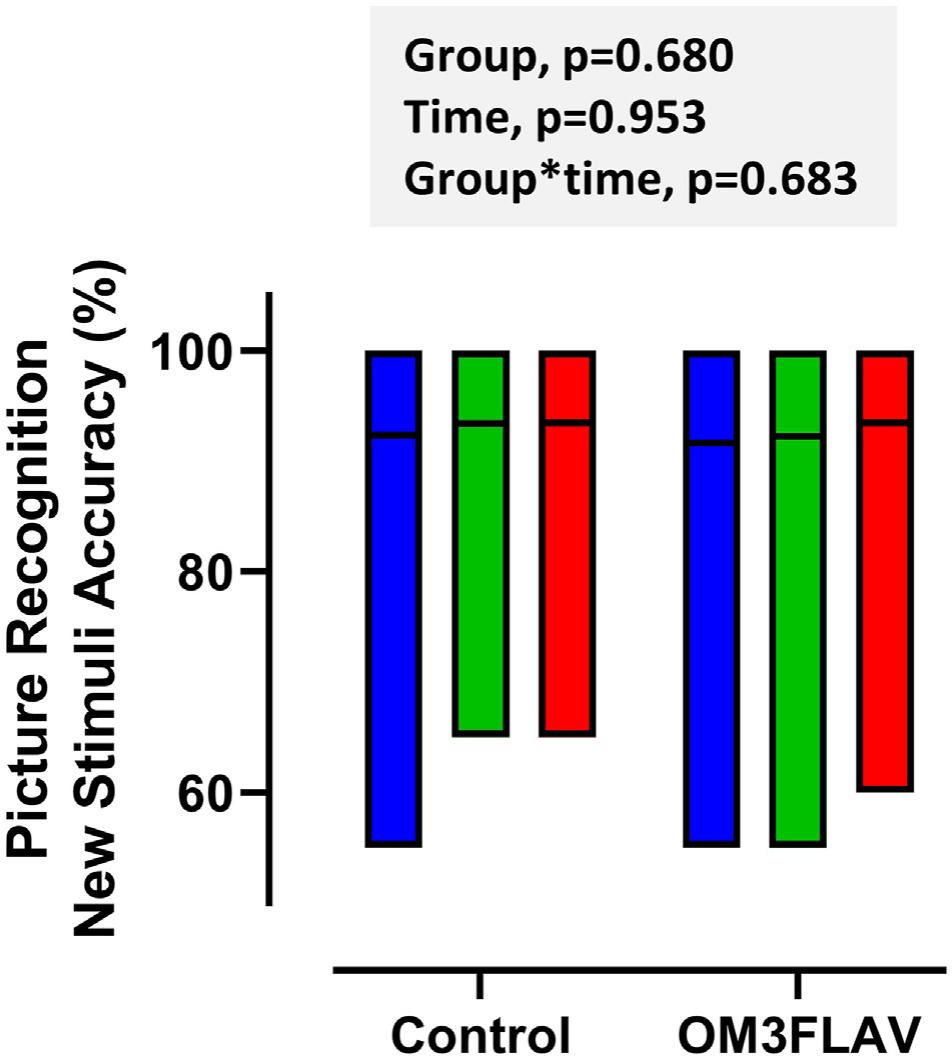
Picture recognition new stimuli accuracy (%) at baseline and following 3 mo or 12 mo of a DHA-rich fish oil and cocoa flavanol mixture (OM3FLAV; *n =* 104 at 3 mo and *n =* 95 at 12 mo) or a control product (*n =* 108 at 3 mo and *n =* 102 at 12 mo). Data are presented as floating bars (min-max) with the horizontal line indicating the mean. Bar colors are as follows: blue (baseline), green (3 mo), and red (12 mo).

No significant impact of intervention on picture recognition performance was evident, with mean (SD) of 85.1 (1.3)% and 85.6 (1.4)% at baseline and 12 mo, respectively (*P =* 0.680, Figure 3), with a prespecified exploratory subgroup analysis indicating no effect of cognitive status (MCI compared with SCI), sex, or *APOE4* carrier status on the response to intervention. The change in picture recognition between baseline and 12 mo was not correlated with the change in EPA or DHA status in plasma NEFA, LPC, or PC fractions or in the 3 FLAV urinary biomarkers, either in the full intervention group or in the OM3FLAV only group (see Supplementary Table 4A and 4B)

### Secondary cognitive outcome responses

There was no significant effect of the intervention on any of the secondary composite or individual cognitive outcomes, with the exception of reaction time variability (*P =* 0.007), executive function (*P <* 0.001), and alertness (*P <* 0.001) (Supplementary Table 5 and Table 3). Post hoc analysis revealed a 4% decreased reaction time variability in the control group, which only reached significance at 12 mo (*P =* 0.022) and only in SCI individuals (*P =* 0.032). For executive function (#), the 3% decrease in the OM3FLAV group by 3 mo (*P =* 0*.*013, 118.5 [25.3] at baseline compared with 113.3 [25.4] at 12 mo) was evident in SCI only (*P =* 0.003) and not observed in the control intervention arm. A decrease in alertness (mm) over time was evident in the OM3FLAV group only (68.5 [14.7] at baseline and 64.0 [17.3] at 12 mo, *P <* 0.001). A significant effect of intervention on these variables was also evident in the intention-to-treat analysis (Table 3).

**TABLE 3:** Cognitive secondary endpoints and response to intervention as per protocol analysis

	Improved performance	0 mo	3 mo	12 mo	*P*_group	*P*_time	*P*_group ×time
**Power of attention (ms)**^1^^,^^2^							
Control (*n =* 101)	Lower	1324 (142)	1322 (133)	1330 (138)	0.058	0.554	0.934
OM3FLAV (*n =* 94)		1343 (158)	1354 (172)	1361 (204)			
**Continuity of attention (#)**^1^^,^^2^							
Control (*n =* 101)	Higher	91.6 (3.5)	92.0 (2.8)	92.0 (2.7)	0.502	0.833	0.921
OM3FLAV (*n =* 94)		91.7 (3.0)	91.8 (2.6)	91.7 (4.5)			
**Reaction time variability (%)**^1^^,^^2^							
Control (*n =* 101)	Lower	51.4 (10.5)	49.6 (9.9)	49.1 (9.0)	0.007	0.624	0.907
OM3FLAV (*n =* 94)		50.4 (10.8)	51.4 (12.0)	51.0 (9.5)			
**Cognitive reaction time (ms)**^1^^,^^2^							
Control (*n =* 101)	Lower	207.1 (72.0)	211.3 (68.7)	209.0 (71.8)	0.880	0.588	0.716
OM3FLAV (*n =* 94)		221.5 (73.3)	219.8 (69.2)	214.2 (71.2)			
**Quality of working memory (SI)**^1^^,^^2^							
Control (*n =* 101)	Higher	1.74 (0.31)	1.81 (0.28)	1.79 (0.29)	0.060	0.659	0.934
OM3FLAV (*n =* 94)		1.73 (0.35)	1.75 (0.35)	1.75 (0.33)			
**Quality of episodic memory (#)**^1^^,^^2^							
Control (*n =* 101)	Higher	174.2 (50.0)	184.2 (51.4)	178.6 (61.7)	0.300	0.747	0.290
OM3FLAV (*n =* 94)		176.0 (56.4)	177.2 (52.0)	181.6 (53.9)			
**Quality of memory (#)**^1^^,^^2^							
Control (*n =* 101)	Higher	347.8 (65.6)	364.5 (69.1)	357.1 (80.0)	0.105	0.536	0.408
OM3FLAV(*n =* 94)		347.3 (72.3)	351.6 (70.7)	355.3 (73.1)			
**Speed of memory (ms)**^1^							
Control (*n =* 101)	Lower	4264 (821)	4183 (924)	4197 (891)	0.094	0.796	0.680
OM3FLAV (*n =* 94)		4332 (1239)	4362 (1170)	4272 (1239)			
**Executive function task (#)**^1^^,^^2^							
Control (*n =* 101)	Higher	118.6 (21.7)	119. 5 (22.5)	119.9 (23.8)	<0.001	0.660	0.758
OM3FLAV (*n =* 94)		118. 5 (25.3)	114.6 (25.8)	113.3 (25.4)			
**Alertness (ms)**^1^							
Control (*n =* 101)	Higher	68.2 (16.0)	69.9 (14.2)	69.5 (14.7)	<0.001	0.381	0.487
OM3FLAV (*n =* 94)		68.5 (14.7)	66.0 (17.9)	64.0 (17.3)			
**MoCA (#)**							
Control (*n =* 101)	Higher	27.2 (1.9)	26.9 (2.2)	27.4 (2.2)	0.401	0.459	0.755
OM3FLAV (*n =* 94)		27.0 (2.1)	27.1 (2.2)	27.5 (2.2)			
**Verbal fluency C1 (#)**							
Control (*n =* 101)	Higher	12.5 (3.4)		12.6 (3.5)	0.724	0.330	0.329
OM3FLAV (*n =* 94)		12.3 (3.5)		12.6 (3.6)			
**Verbal fluency C3a (#)**							
Control (*n =* 101)	Higher	11.5 (3.6)		12.0 (3.6)	0.812	0.215	0.092
OM3FLAV (*n =* 94)		12.1 (3.3)		11.9 (4.3)			
**iPosition (pixels) (Original misplacement)**							
Control (*n =* 100)	Lower	241.9 (72.0)	237.9 (81.4)	231.1 (77.8)	0.335	0.135	0.151
OM3FLAV (*n =* 90)		250.8 (69.6)	235.7 (71.1)	236.3 (78.1)			
**iPosition (#) Accuracy single item placement**							
Control (*n =* 100)	Higher	2.45 (0.61)	2.49 (0.65)	2.51 (0.61)	0.998	0.014	0.427
OM3FLAV (*n =* 90)		2.37 (0.63)	2.44 (0.62)	2.45 (0.66)			

Data are mean (SD); ANCOVA using mixed models for repeated measures. TREATMENT was fitted as a fixed between-group factor (2 levels: intervention or control group). Change from baseline was calculated for all outcome variables (ie, 3 mo-0 mo and 12 mo-0 mo), and VISIT was fitted as a fixed repeated factor (2 levels: 3 and 12 mo). BASELINE (predose scores), study site, BMI, and years of education were added as covariates in the final model.

1 part of the CDR test battery

2 a composite measure; Verbal Fluency C1- condition 1, letter fluency, total correct and scaled for age; Verbal Fluency C3a- condition 3, category switching, total correct responses and scaled for age; # Number of items. CDR, Cognitive Drug Research; OM3FLAV, omega-3 polyunsaturated fatty acids (EPA+DHA; OM3) + cocoa flavan-3-ols (FLAV).

### Brain volumes

**FIGURE 4:**
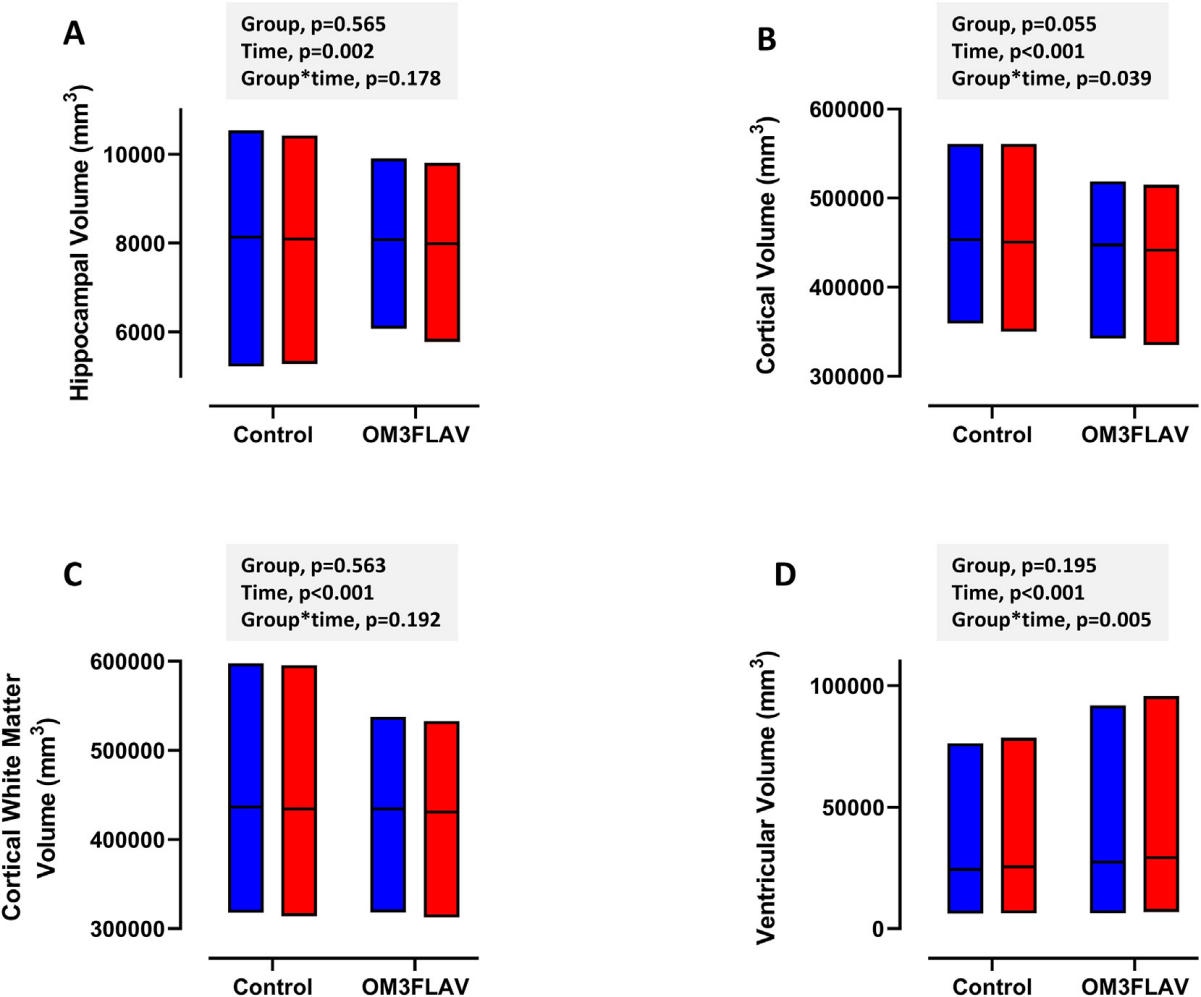
Structural MRI assessment of (A) hippocampal volume; (B) cortical volume; (C) cortical white matter volume; and (D) ventricular volume following 12-mo consumption of a DHA-rich fish oil and cocoa flavanol mixture (OM3FLAV; *n =* 48) or a control product (*n =* 62). Data are presented as floating bars (min-max) with the horizontal line indicating the mean. Bar colors are as follows: blue (baseline) and red (12 mo).

The baseline MRI data in the group as a whole (*n =* 140) and according to center is given in Supplementary Table 6, with no difference between center for brain volume (*P =* 0.256-0.934). A significant 0.7% to 1.0% decrease in volume in all 3 regions over the 12-mo period was evident in the group as a whole (*P <* 0.001) (Figure 4). For cortical volume (mm^3^), a group×time effect was evident (*P =* 0.039*)*, with a greater decrease in the OM3FLAV (446,813 [2850] at baseline, 441,087 [2850] at 12 mo) compared with the control (453,634 [3239] at baseline, 450,829 [3239] at 12 mo). For ventricular volume, a greater increase was evident in the OM3FLAV group (*P =* 0.005*).* No effect of treatment was evident for the hippocampal (*P =* 0.178*)* or cortical white matter (*P =* 0.192*)* volumes.

### Plasma biochemistry

The intervention decreased plasma triglyceride levels by 22% in the OM3FLAV group by 3 mo, with no change in the control group (*P <* 0.001, Table 4). Relative to the control, OM3FLAV resulted in a significant increase in total cholesterol (*P =* 0.008), HDL (*P <* 0.001), and glucose (*P =* 0.008) of 2.8%, 10.2%, and 2.9% respectively. No impact of intervention was observed for BDNF concentrations (*P =* 0.265).

**TABLE 4:** Plasma biochemistry at baseline and in response to intervention

	0 mo	3 mo	12 mo	*P*_group	*P*_time	*P*_group×time
**BDNF (pg/ml)**						
Control (n = 87)	19,596 (4685)	19,977 (4995)	20,270 (5088)	0.198	0.140	0.265
OM3FLAV (n = 99)	18,403 (4685)	18,525 (4897)	18,381 (4884)			
**Triglycerides (mmol/l)**						
Control (n = 101)	1.14 (0.40)	1.14 (0.46)	1.18 (0.51)	<0.001	0.458	0.459
OM3FLAV (n = 93)	1.30 (0.67)	1.02 (0.39)	1.05 (0.48)			
**Cholesterol (mmol/l)**						
Control (n = 101)	5.11 (0.98)	5.22 (1.01)	5.29 (0.96)	0.008	0.537	0.796
OM3FLAV (n = 93)	5.17 (1.04)	5.43 (1.14)	5.47 (1.18)			
**HDL-Cholesterol (mmol/l)**						
Control (n = 101)	1.51 (0.46)	1.56 (0.47)	1.56 (0.48)	<0.001	0.869	0.531
OM3FLAV (n = 93)	1.41 (0.42)	1.59 (0.50)	1.60 (0.50)			
**HDL-Cholesterol: Cholesterol**						
Control (n = 101)	0.298 (0.084)	0.302 (0.083)	0.299 (0.085)	<0.001	0.651	0.386
OM3FLAV (n = 93)	0.276 (0.081)	0.297 (0.084)	0.299 (0.090)			
**Glucose (mmol/l)**						
Control (n = 101)	5.25 (0.91)	5.38 (1.11)	5.33 (0.97)	0.008	0.559	0.490
OM3FLAV (n = 93)	5.18 (0.70)	5.42 (0.77)	5.41 (0.95)			

Data are mean (SD); ANCOVA using mixed models for repeated measures, with change from baseline (ie, 3 mo-0 mo and 12 mo-0 mo) fitted as a fixed repeated factor (2 levels: 3 and 12 mo), and center, age, sex, and BMI added as covariates. BDNF, brain-derived neurotrophic factor; HDL, high density lipoprotein; OM3FLAV, omega-3 polyunsaturated fatty acids (EPA+DHA; OM3) + cocoa flavan-3-ols (FLAV).

### Dietary data

Habitual intakes of oily fish (main dietary source of EPA and DHA), total flavonoids and flavan-3-ol were 0.724 portions per week, 842 mg/d and 193 mg/d respectively, with no baseline difference between groups (Table 5). Habitual intake by trial site is given in Supplementary Table 7, with the higher total flavonoid and flavan-3-ol intake in UK participants driven by higher tea intake. Higher consumption of carbohydrate was also observed in UK. No group × time interaction was evident for habitual diet with the exception of a reduction in total calorie (*P =* 0.043) and protein (*P =* 0.010) intake in the control groups relative to the OM3FLAV from baseline to 12m.

**TABLE 5:** Nutrient intake (derived from the Food Frequency Questionnaire) at baseline and 12 mo

	0 mo	12 mo	*P*_group×time
**Total Energy (kcal/d)**			
Control (*n =* 99)	2009 (747)	1784 (652)	0.043
OM3FLAV (*n =* 91)	1958 (678)	1881 (615)	
**Fat (g/d)**			
Control (*n =* 99)	80.1 (32.6)	71.7 (28.1)	0.192
OM3FLAV (*n =* 91)	80.3 (32.7)	76.2 (29.6)	
**Carbohydrate (g/d)**			
Control (*n =* 99)	234 (103)	206 (86)	0.069
OM3FLAV (*n =* 91)	221 (85)	211 (76)	
**Protein (g/d)**			
Control (*n =* 99)	87.6 (29.7)	78.8 (28.8)	0.010
OM3FLAV (*n =* 91)	87.6 (25.4)	88.2 (30.2)	
**Total sugars (g/d)**			
Control (*n =* 99)	122 (52)	109 (50)	0.117
**OM3FLAV (*n =* 91)**	112 (49)	108 (44)	
**Oily Fish (portions/wk)**			
Control (*n =* 99)	0.67 (0.67)	0.76 (0.83)	0.567
**OM3FLAV (*n =* 90)**	0.76 (0.79)	0.77 (0.88)	
**Total Flavonoids (mg/d)**			
Control (*n =* 99)	845 (460)	779 (453)	0.908
OM3FLAV (*n =* 91)	840 (449)	779 (436)	
**Flavan-3-ols (mg/d)**			
Control (*n =* 99)	177 (113)	163 (107)	0.313
OM3FLAV (*n =* 91)	210 (214)	176 (153)	

Data are mean (SD); Intervention Group × Time (baseline, 12 mo) interaction derived from Repeated Measures ANOVA (*P* group×time). Statistical tests for oily fish run on Log transformed data (Ln(portions+1)), raw means reported for interpretability.

Analyses were run for the full sample with complete data (results shown) and repeated excluding those reporting total energy expenditure less than 1.1 times their basal metabolic rate (BMR). When excluding underreporters, results were unchanged apart from the following: Carbohydrate, groups differed at baseline (*P =* 0.025), interaction remains nonsignificant (*P =* 0.071); Total Sugars, groups differ at baseline (*P =* 0.013), interaction remains nonsignificant (*P =* 0.179).

OM3FLAV, omega-3 polyunsaturated fatty acids (EPA+DHA; OM3) + cocoa flavan-3-ols (FLAV).

## Discussion

The current study provides the first long-term data to suggest that supplementation with long-chain ω-3 fatty acids and FLAVs over 1 y does not improve cognitive function or brain volumes in individuals with subjective and objective cognitive impairments. The combined dietary intervention resulted in the expected several-fold increases in plasma EPA, DHA, and urinary flavanol metabolite concentrations.

This null finding raises several questions regarding the intervention, methodology, and public health implications, which are considered in the following sections. One possibility is that the cognitive battery employed is not sensitive to changes relating to dietary intervention [49]. However, the CDR battery has been used in numerous drug trials and in a number of acute and chronic food interventions. It is known to be sensitive to bidirectional change in the target population, having detected intervention effects in cognitively intact older cohorts as well as those with SCI and MCI [50,51]. A related point is that attrition was higher than anticipated, resulting in 95 and 102 completers in the OM3FLAV and placebo groups, respectively. Although this raises the possibility that that study was underpowered, the lack of any evidence of a OM3FLAV mediated benefit for the range of cognitive outcomes included, as reflected in the *P* values (eg, *P =* 0.68 for the primary outcome), indicates that this is a genuine null finding.

A second possibility is that the 1-y intervention period was not long enough for the neurophysiological changes associated with PUFAs and FLAVs to become evident. The prodrome of dementia is 20 to 30 y or longer. In prospective cohort studies, positive associations between EPA+DHA and FLAV intakes and cognitive and brain structure represent the benefit of long-term, often decades of habitual dietary exposure [5,8,22].

The inclusion of those with SCI and MCI profiles was chosen to increase the translatability of the findings (allowing an exploratory analysis to be conducted as to the most effective window of intervention opportunity). It may be considered as a study limitation, however, potentially increasing the heterogeneity of the response to intervention and thereby decreasing the likelihood of detecting a significant effect of intervention. Although the study is not powered to formally address this issue, we note that there were group differences, specifically for 3 cognitive tests in SCI (reaction time variability, quality of memory, and executive function score) and 2 in MCI, power of attention and picture recognition new stimuli speed). There may be value in targeting more homogenous cohorts in future studies.

The lack of effect of OM3FLAV in CANN is consistent with previous interventions that have supplemented with EPA and/or DHA in isolation, as recently reviewed [22]. In the Multidomain Alzheimer Preventive Trial, which recruited nondemented older adults with subjective memory complaints consuming 1025 mg EPA+DHA for 3 y, had no effect on a compositive cognitive Z-score [24]. As the brain DHA half-life is estimated to be 2.5 y [24], it may be that supplementation periods of more than 1 y are needed to fully realize any cognitive benefits associated with EPA and DHA enrichment of brain neuronal and glial cells, such as effects on dendrite outgrowth and spine density, synaptic function, and neuroinflammation. Cocoa FLAV effects have been observed acutely [52], albeit in more mentally challenging tasks than the ones used here, and in chronic studies within 8 to 12 wk [18,20].

The impact of intervention length on neurocognitive benefits is evidenced by comparing the 24-mo and 36-mo findings from the LipiDiDiet study [53,54]. At 24 mo, although an effect of intervention on secondary outcomes was observed (hippocampal volume and CDR score), no effect on the primary outcome, the neurocognitive test battery performance, was evident. By 36 mo, the intervention had significantly increased the neurocognitive test battery score relative to the control group.

A lower calorie and protein intake in the control group relative to OM3FLAV at 12 mo may have resulted in lower body weight, which could contribute to improved cognition relative to OM3FLAV [55]. However, given that weight was not included as a secondary outcome, which is a recognized study limitation, we cannot say with any certainty that this modest deficit in energy intake contributed to intergroup differences in cognitive performance and cannot be ruled out as a contributing factor. In addition, there were some differences in habitual carbohydrate and flavonoid intake between the study sites; however, this is highly unlikely to have impacted on the trial findings, given the randomized controlled nature of the intervention across sites, with site added as a covariate to all analyses.

There are a number of considerations regarding the intervention itself. One possibility is that there were changes to the interventions during the trial that reduced their functional efficacy. The shelf life of fish oils (FOs) is dependent on certain storage microenvironment conditions to prevent lipid peroxidation. The FO capsules were enriched with mixed tocopherol (3.8 mg/g oil) to prevent any loss of EPA and DHA content. Intervention product fatty acid and FLAV analysis at 1 y indicated no loss of EPA, DHA or flavan-3-ol levels. The FLAV-rich chocolate chips provided 30 mg of caffeine and 262 mg theobromine daily relative to 5.4 mg and 64 mg daily (Supplementary Table 1) in the control intervention. Given that an average cup of filter coffee contains about 100 mg of caffeine, a daily 25-mg deficit between intervention groups is unlikely to explain the tendency toward a reduced performance for a small number of cognitive outcomes in the OM3FLAV group. Cocoa and chocolate are the main dietary sources of theobromine. Although average habitual theobromine intake and its potential cognitive modulating properties are largely unknown [56], a long term higher intake could potentially have negative neurophysiological properties [57]. Additionally, the control and active chocolate chip products, although isocaloric and well matched for fat and total carbohydrate content, differed with respect to sugars and protein provision per day (see Supplementary Table 1). This limitation is partly due to the well-recognized food technological difficulties of producing matched products when conducting whole food interventions. However, the higher protein and sugar content in the control chocolate chip was negated by a reduced daily habitual dietary protein and sugar intake in the control group over the 12-mo intervention period (Table 5).

Of the 11 broad secondary outcomes, performance on one measure of Executive Function (EF) declined in the OM3FLAV group, which was unexpected. The other widely used measures of EF, Verbal Fluency, and Category Switching (measured at baseline and 12 mo) showed no treatment effects. Additionally, EF is generally accepted to rely on prefrontal cortex activity, and we did not find evidence of differential effects on this area (with the caveat that it was not a specific focus of the study).

We can be relatively confident that the interventions were consumed as directed. Compliance checks based on intervention returns were good (>95% in both arms), which were supported by plasma and urine fatty acid and flavonoid metabolite enrichment, respectively. Importantly, changes to these biomarkers did not correlate with changes in cognitive function, suggesting that there were not individual differences in intervention response that were related to detectable cognitive benefits.

No effect of intervention was evident for hippocampal volume, which was the preregistered secondary outcome for structural MRI. The 0.8% decline in volume of this region over 12 mo in the group as a whole is in line with or potentially lower than would be expected in a mixed SCI/MCI group [58,59]. It is noteworthy that the significant intervention effects in select cognition measures in favor of the control group was associated with a lower cortical volume loss following control versus OM3FLAV intervention (−0.6% compared with −1.3%) and smaller increase in ventricular volume (4.4% and 6.8%). Although directly comparable normative data for a mixed SCI/MCI group are not readily available, the magnitude of change over 12 mo in cortical volume is within the expected range from the published literature [59,60], with a trend toward a lower cortical volume at baseline in the OM3FLAV group potentially contributing to the greater loss in volume over the intervention period.

Our lack of benefit of OM3FLAV is consistent with the only other randomized controlled trial (RCT) to cosupplement flavonoids (anthocyanins from blueberry powder [BB] and FO providing 1.6 g EPA and 0.8 g DHA per day) for 24 wk in an SCI population [61]. In this 4-arm parallel study, the FO and BB were fed either in isolation or combined and compared to a control intervention. Although the BB and FO groups reported fewer cognitive symptoms and the BB group showed improved memory discrimination, the combined BB+FO intervention was not associated with cognitive enhancement as anticipated. This raises the possibility that flavonoid and ω-3 PUFA combinations may be mutually antagonistic regarding certain processes underlying cognitive performance.

Another possible factor that may contribute to these findings is that the intervention was associated with higher glucose levels, which are known to contribute to cognitive decline via central insulin sensitivity [62]. However, the effect-size OM3FLAV effect was modest (2.9% increase over 12 mo) and therefore unlikely to underpin the null findings. Consistent with most previous RCTs targeting cognition, and the fact that brain DHA levels are >250 higher than EPA (152), the FO capsules fed in CANN were DHA rich (DHA:EPA, 2.75). EPA does enter the brain with uptake efficiencies equivalent to DHA but is thought to be rapidly metabolized following entry, although concentrations of EPA are higher than DHA in microglial cells [63]. The impact of EPA in cognition is being increasingly recognized [64]. Future interventions should not only consider what dose but also what DHA:EPA ratio of the supplement used.

In conclusion, 1 y intervention with EPA+DHA and cocoa FLAVs did not improve cognition or protect the brain against atrophy in older adults with evidence of memory deficits. However, based on strong prospective cohort evidence of an association between long-term fish/EPA+DHA and flavonoid intake and status and cognitive well-being, and evidence of subgroup variability of response in the current trial, the findings do not preclude modest benefits of supplementation in responsive subgroups. Given the complexity of neuropathological processes underpinning cognitive decline and dementia risk, it is emerging that, at a population level, multidomain [65], multinutrient [54], or whole diet approaches [66] may be needed to positively impact the cognitive trajectory in the medium term (months to 3 y).

## Data Availability

Data described in the manuscript, code book, and analytic code will be made available upon request pending application and approval.

## Appendix A Supplementary data

Multimedia component1 Multimedia component1
