# Supplementary material for: A combined DHA-rich fish oil and cocoa flavanols intervention does not improve cognition or brain structure in older adults with memory complaints: results from the CANN randomized, controlled parallel-design study
Source: Am J Clin Nutr. 2023 Jun 12;118(2):369–81. doi: 10.1016/j.ajcnut.2023.06.008 (PMC10447509; doi:10.1016/j.ajcnut.2023.06.008)
Supplement: Multimedia component1 [file mmc1.docx]

**A combined DHA-rich fish oil and cocoa flavanols intervention does not improve cognition or brain structure in older adults with memory complaints: Results from the CANN randomized, controlled parallel-design study**

David Vauzour^1†^*, Andrew Scholey^2†^*, David J. White^2^, Neal J Cohen^3^, Aedín Cassidy^1,4^, Rachel Gillings^1^, Michael A Irvine^1^, Colin D. Kay^5^, Min Kim^6^, Rebecca King^2^, Cristina Legido-Quigley^6^, John F. Potter^1^, Hilary Schwarb^3^, Anne-Marie Minihane^1,7^*

^1^ Norwich Medical School, University of East Anglia (UEA), Norwich, UK

^2^ Centre for Human Psychopharmacology, Swinburne University, Australia

^3^ Beckman Institute for Advanced Science and Technology, University of Illinois at Urbana-Champaign, Urbana, Illinois, USA.

^4^ Institute for Global Food Security, Queen's University Belfast, Belfast, Northern Ireland

^5^ Plants for Human Health Institute, Food Bioprocessing and Nutrition Sciences Department, North Carolina State University, North Carolina Research Campus, 600 Laureate Way, Kannapolis, North Carolina 28081, USA

^6^ Translational and Clinical Chemistry, Franklin-Wilkins Building, Stamford Street, Kings College London, London, UK

^7^ Norwich Institute of Healthy Ageing (NIHA), UEA, Norwich, UK

***Correspondence:** Dr David Vauzour (D.Vauzour@uea.ac.uk), Pr Anne Marie Minihane (A.Minihane@uea.ac.uk) and Pr Andrew Scholey (andrew@scholeylab.com).

**^†^** These authors share first co-authorship.

**Supplementary Table 1: Chocolate chips composition**

|  | **OM3FLAV** | **CONTROL** |
| --- | --- | --- |
| Packet size, g | **33** | **33** |
| Cocoa Flavanols (DP 1-10) mg | **508** | **38** |
| Epicatechin, mg | **80** | **7** |
| Catechin, mg | **26** | **4** |
| Dimers-decamers, mg | **402** | **27** |
| Calories | **158** | **160** |
| Total fat, g | **12** | **7.5** |
| Saturated fat, g | **7** | **7** |
| Cholesterol, mg | **5** | **0.05** |
| Sodium, mg | **3** | **14** |
| Total Carbohydrates, g | **17** | **24** |
| Sugars, g | **10.6** | **23.0** |
| Protein, g | **2.6** | **0.6** |
| Caffeine, mg | **30.0** | **5.4** |
| Theobromine, mg | **262** | **64** |
| Potassium, mg | **241** | **97** |

**Supplementary Table 2: *APOE* genotypes of participants at baseline (total number and according to cognitive status (e.g., SCI versus MCI)**

| *APOE* Genotype | Total | SCI | MCI |
| --- | --- | --- | --- |
| E2/E2 | 3 | 0 | 3 |
| E2/E3 | 27 | 18 | 9 |
| E2/E4 | 5 | 5 | 0 |
| E3/E3 | 142 | 81 | 61 |
| E3/E4 | 65 | 38 | 27 |
| E4/E4 | 4 | 0 | 4 |

**Supplementary Table 3: Detail of the metabolites analysed in urine. Quantification was carried out using reference standards or by use of structurally related compounds.**

| **Full Name** | **CAS** | **Reference Standard** | **Relative Quantifier** |
| --- | --- | --- | --- |
| 7-methyxanthine | 552-62-5 | ✓ | reference standard |
| 3-methylxanthine | 1076-22-8 | ✓ | reference standard |
| 1-methyxanthine | 6136-37-4 | ✓ | reference standard |
| 1,7-Dimethylxanthine | 611-59-6 | ✓ | reference standard |
| 1,3-Dimethylxanthine | 58-55-9 | ✓ | reference standard |
| 1,3,7-Trimethylxanthine | 58-08-2 | ✓ | reference standard |
| 3,4-dihydroxyphenyl-gamma-valerolactone | 191666-22-5 | ✓ | reference standard |
| 3-hydroxyphenyl-gamma-valerolactone-4-sulfate | N/A | ✓ | reference standard |
| Epicatechin | 490-46-0 | ✓ | reference standard |
| 5-(4'-Hydroxyphenyl)-gamma-valerolactone | 871329-30-5 | ✓ | reference standard |
| 5-(3'-Hydroxyphenyl)-gamma-valerolactone | 21618-91-7 | ✓ | reference standard |
| 5-(4-hydroxyphenyl)valeric acid | 4654-08-4 | ✓ | reference standard |
| Hydroxyphenyl)-valeric acid-sulfate | x | x | 3-hydroxyphenyl-gamma-valerolactone-4-sulfate |
| Hydroxyphenyl)-valeric acid-sulfate | x | x | 3-hydroxyphenyl-gamma-valerolactone-4-sulfate |
| Hydroxyphenyl)-valeric acid-sulfate | x | x | 3-hydroxyphenyl-gamma-valerolactone-4-sulfate |
| Hydroxyphenyl)-valeric acid-sulfate | x | x | 3-hydroxyphenyl-gamma-valerolactone-4-sulfate |
| Hydroxyphenyl)-valeric acid-sulfate | x | x | 3-hydroxyphenyl-gamma-valerolactone-4-sulfate |
| Epicatechin sulfoglucuronide | x | x | Epicatechin |
| Epicatechin sulfoglucuronide | x | x | Epicatechin |
| Epicatechin sulfoglucuronide | x | x | Epicatechin |
| 5-(Phenyl)-γ-valerolactone-3-sulfate-4-*O*-glucuronide | x | x | 3-hydroxyphenyl-gamma-valerolactone-4-sulfate |
| 5-(3'-Hydroxyphenyl)-γ-valerolactone-4'-*O*-glucuronide | x | x | 3-hydroxyphenyl-gamma-valerolactone-4-sulfate |
| 5-(phenyl)-gamma-valerolactone-3-*O*-glucuronide | x | x | 3-hydroxyphenyl-gamma-valerolactone-4-sulfate |
| 5-(phenyl)-gamma-valerolactone-3-*O*-glucuronide | x | x | 3-hydroxyphenyl-gamma-valerolactone-4-sulfate |
| 5-(phenyl)-gamma-valerolactone-3-*O*-glucuronide | x | x | 3-hydroxyphenyl-gamma-valerolactone-4-sulfate |
| 5-(phenyl)-gamma-valerolactone-3-*O*-glucuronide | x | x | 3-hydroxyphenyl-gamma-valerolactone-4-sulfate |
| 5-(phenyl)-gamma-valerolactone-4-*O*-glucuronide | x | x | 3-hydroxyphenyl-gamma-valerolactone-4-sulfate |
| 5-(phenyl)-gamma-valerolactone-4-*O*-glucuronide | x | x | 3-hydroxyphenyl-gamma-valerolactone-4-sulfate |
| 5-(phenyl)-gamma-valerolactone-4-*O*-glucuronide | x | x | 3-hydroxyphenyl-gamma-valerolactone-4-sulfate |
| 5-(4',5'-dihydroxyphenyl)-gamma-valerolactone-O-sulfate | x | x | 3-hydroxyphenyl-gamma-valerolactone-4-sulfate |
| 5-(4',5'-dihydroxyphenyl)-gamma-valerolactone-O-sulfate | x | x | 3-hydroxyphenyl-gamma-valerolactone-4-sulfate |
| Hydroxyphenyl-γ-valerolactone-sulfate | x | x | 3-hydroxyphenyl-gamma-valerolactone-4-sulfate |
| Hydroxyphenyl-γ-valerolactone-sulfate | x | x | 3-hydroxyphenyl-gamma-valerolactone-4-sulfate |
| Epicatechin-sulfate | x | x | Epicatechin |
| Epicatechin-sulfate | x | x | Epicatechin |
| 5-(3-hydroxyphenyl)valeric acid | x | x | 5-(4-hydroxyphenyl)valeric acid |
| 5-(hydroxyphenyl)valeric acid | x | x | 5-(4-hydroxyphenyl)valeric acid |
| 5-(Phenyl)-γ-valerolactone-3'-sulfate | x | x | 3-hydroxyphenyl-gamma-valerolactone-4-sulfate |
| Epicatechin-*O*-glucuronide | x | x | Epicatechin |
| Epicatechin-*O*-glucuronide | x | x | Epicatechin |
| 5-(dihydroxyphenyl)-gamma-valerolactone | x | x | 3,4-dihydroxyphenyl-gamma-valerolactone |
| 3'-*O*-Methyl-epicatechin-sulfate | x | x | Epicatechin |
| 3'-*O*-Methyl-epicatechin-sulfate | x | x | Epicatechin |

**Supplementary Table 4a: Correlation between the 0-12m change in EPA and DHA in various lipid fractions with the change in Picture Recognition over the intervention period in the group as a whole (n=190) or OM3FLAV only (n=90)**

|  | NEFA_EPA | NEFA_DHA | PC (36:5) | PC (38:6) | PC (40:6) | LPC-EPA | LPC-DHA |
| --- | --- | --- | --- | --- | --- | --- | --- |
| All  r^2^  P | -0.055  0.454 | -0.096  0.188 | -0.053  0.472 | -0.043  0.556 | -0.044  0.546 | 0.079  0.276 | 0.073  0.316 |
| OM3FLAV  r^2^  P | 0.069  0.518 | 0.018  0.867 | -0.042  0.692 | 0.018  0.864 | 0.029  0.784 | 0.122  0.253 | 0.117  0.270 |

R^2^ is the Pearson’s correlation coefficient; DHA, docosahexaenoic acid; EPA, eicosapentaenoic acid; LPC, lysophosphatidylcholine; NEFA, non-esterified fatty acids; PC-phosphatidylcholine

PC.36:5 (16:0/20:5, 16:1/20:4, 18:2/18:3), PC.38:6 (16:0/22:6, 18:1/20:5, 18:2/20:4), PC.40:6 (18:0/22:6, 18:1/22:5, 18:2/22:4, 20:2/20:4)

**Supplementary Table 4b: Correlation between the 0-12m change in urinary FLAV metabolites with the change in Picture Recognition over the intervention period in the group as a whole (n=183) or OM3FLAV only (n=90)**

|  | 5-(Phenyl)-γ-valerolactone-3'-sulfate | Hydroxyphenyl-γ-valerolactone-sulfate | 5-(3'-Hydroxyphenyl)-γ-valerolactone-4'-O-glucuronide |
| --- | --- | --- | --- |
| All  r^2^  P | -0.142  0.055 | -0.004  0.955 | -0.003  0.969 |
| OM3FLAV  r^2^  P | -0.199  0.066 | -0.006  0.957 | -0.006  0.956 |

R^2^ is the Pearson’s correlation coefficient,

**Supplementary Table 5: Select cognitive outcome response according to cognitive status at baseline**

| Study Measure | Main Effect of Treatment  p-value | | |
| --- | --- | --- | --- |
|  | **Whole Population^a^**  **(n=246)** | **SCI^a^**  **(n=142)** | **MCI^a^**  **(n=104)** |
| Picture Recognition New Stimuli Accuracy | 0.680 | 0.773 | 0.977 |
| Picture Recognition Original Stimuli Accuracy | 0.661 | 0.277 | 0.354 |
| Picture Recognition New Stimuli Speed | 0.134 | 0.957 | 0.034 |
| Picture Recognition Original Stimuli Speed | 0.751 | 0.384 | 0.315 |
| Power of Attention | 0.058 | 0.927 | 0.025 |
| Continuity of Attention | 0.502 | 0.334 | 0.935 |
| Reaction Time Variability | 0.007 | 0.032 | 0.201 |
| Quality of Working Memory | 0.060 | 0.083 | 0.440 |
| Quality of Episodic Memory | 0.300 | 0.127 | 0.370 |
| Quality of Memory | 0.105 | 0.033 | 0.637 |
| Speed of Memory | 0.094 | 0.085 | 0.403 |
| Executive Function Score | <0.001 | <0.001 | 0.405 |

^a^ P-values, ANCOVA using mixed models for repeated measures. TREATMENT was fitted as a fixed between group factor (2 levels: intervention or control group). Change from baseline was calculated for all outcome variables (i.e. 3m-0m and 12m-0m) and VISIT was fitted as a fixed repeated factor (2 levels: 3 & 12 months). BASELINE (pre-dose scores), study site, BMI and years of education were added as co-variates in the final model. MCI, mild cognitive impairment; SCI, subjective cognitive impairment

**Supplementary Table 6: Baseline characteristics of participants who underwent MRI scanning in the group as a whole and according to centre**

|  | Overall  (n=140) | Norwich  (n=71) | Melbourne  (n=69) | P values^a^ |
| --- | --- | --- | --- | --- |
| age (years) | 65.3 (6.7) | 65.6 (6.7) | 65.1 (6.8) | 0.711 |
| Sex, M/F (%F) | 53/87 (62) | 28/43 (61) | 25/44 (64) | 0.414 |
| Education (years) | 14.2 (3.5) | 13.3 (2.8) | 15.2 (3.9) | 0.002 |
| SCI/MCI (%MCI) | 69/71 (51) | 37/34 (48) | 32/37 (54) | 0.305 |
| Hippocampal volume (cm^3^) | 8007 (979) | 8100 (1005) | 7912 (950) | 0.256 |
| Cortical volume (cm^3^) | 446961 (44605) | 444095 (42327) | 449910 (46961) | 0.443 |
| Cortical white matter volume (cm^3^) | 429454 (56383) | 425155 (53065) | 433877 (59671) | 0.363 |
| Ventricular volume (cm^3^) | 25472 (15704) | 25363 (16145) | 25583 (15354) | 0.934 |

Data are mean (SD) or as stated n (%)

^a^ Independent t-test for continuous data and Chi-square test for nominal data

MCI, mild cognitive impairment; SCI, subjective cognitive impairment

**Supplementary Table 7: Baseline nutrient intake (derived from the Food Frequency Questionnaire) by Country (Trial site)**

|  | UEA; Norwich, UK (n=123) | SUT; Melbourne, Australia (n=67) | P_baseline |
| --- | --- | --- | --- |
| Total Energy (kcal/d) | 2057 (726) | 1851 (674) | 0.057 |
| Fat (g/d) | 82.01 (32.73) | 76.86 (32.29) | 0.299 |
| Carbohydrate (g/d) | 238.92 (98.87) | 206.16 (83.11) | **0.022** |
| Protein (g/d) | 89.80 (24.99) | 83.45 (31.85) | 0.131 |
| Total Sugars (g/d) | 122.22 (51.12) | 108.71 (49.29) | 0.080 |
| Oily Fish (portions /week) | 0.75 (0.76) | 0.67 (0.70) | 0.459 |
| Total Flavonoids (mg/d) | 956.24 (450.74) | 633.17 (378.68) | **<0.001** |
| Flavan-3-ols (mg/d) | 222.98 (189.56) | 137.00 (105.56) | **<0.001** |

Data are mean (SD); Between groups T-test comparing baseline nutrient intake between trial sites for sample with nutrient data at baseline and post-treatment (n=190). Statistical tests for Oily Fish run on Log transformed data (Ln(portions+1)), raw means reported for interpretability.
